# Association of food insecurity with diet quality and anthropometric measurements among American elderly: results from 2017 to 2020 National Health and Nutrition Examination Survey

**DOI:** 10.1017/S0007114525103504

**Published:** 2025-06-28

**Authors:** Xiuhong Wang, Hui Cao, Xuanlan Wu, Yan Xia, ShengJun Wu

**Affiliations:** 1 Department of Clinical Laboratory, Sir Run Run Shaw Hospital, Zhejiang University School of Medicine, Hangzhou 310018, Zhejiang, People’s Republic of China; 2 Department of Hematology, Sir Run Run Shaw Hospital, Zhejiang University School of Medicine, Hangzhou 310016, People’s Republic of China

**Keywords:** Food insecurity, Diet quality, Obesity, Older adults, NHANES

## Abstract

Food insecurity (FIS) is a critical public health issue, particularly among older adults. This study investigates the association between FIS with diet quality and anthropometric indices in the US older adults. A cross-sectional analysis was conducted using NHANES data from 2017 to 2020, involving 2592 participants aged ≥ 60 years. FIS was assessed using the USDA Household Food Security Survey Module. Diet quality was assessed using the Healthy Eating Index (HEI)-2020 and adherence to Mediterranean diet (MedDiet) score. Anthropometric measures were calculated following standardised protocols. Multivariable logistic regression models, adjusted for demographic, socio-economic and behavioural factors examined the association between FIS and the higher quartile and tertile of anthropometric and diet quality indices, respectively. Of the participants, 27·4 % experienced FIS. FIS participants were younger and had lower education and income levels compared with FS individuals (*P* < 0·05). In the adjusted model, FIS was associated with lower adherence to both the Mediterranean Diet (OR: 0·48, 95 % CI: 0·31, 0·67) and HEI-2020 (OR: 0·61, 95 % CI: 0·37, 0·84), indicating poorer diet quality in older adults. In adjusted analyses, FIS was significantly associated with higher A Body Shape Index quartiles (Q3: OR: 1·44, 95 % CI: 1·06, 1·95; Q4: OR: 1·46, 95 % CI: 1·07, 2·01), the waist-to-hip ratio (Q4: OR: 1·44, 95 % CI: 1·01, 2·06) and the Conicity index (Q4: OR: 1·36, 95 % CI: 1·02, 1·81). FIS in older adults is associated with unfavourable diet quality and body composition patterns, particularly central obesity measures. Addressing FIS may mitigate health risks related to obesity and its complications.

Food security, defined as ‘access by all people at all times to enough food for an active, healthy life’, is critical for maintaining health and quality of life^(1)^. Conversely, food insecurity (FIS) poses significant risks to health, particularly among vulnerable populations like the elderly^(2)^. According to early reports by the US Department of Agriculture for 1999, 5·8 % of US households with elderly members face difficulties meeting basic food needs^(3)^. More recent data from 2021 reveal that FIS continues to affect millions (7·1 % of US households with elderly members), emphasising its relevance as a public health issue^(4)^.

Older adults are particularly susceptible to the detrimental effects of FIS, which can exacerbate chronic diseases, frailty and emotional and economic burdens^(5)^. Previous studies have linked FIS to lower diet quality using earlier national datasets and dietary indices (e.g. the Healthy Eating Index (HEI)-2015, the Alternate Healthy Eating Index (AHEI)-2010 and the Mediterranean Diet (MedDiet) scores) among various populations, including older adults^(6,7)^. However, few have utilised updated measures such as the HEI-2020, aligned with the 2020–2025 Dietary Guidelines for Americans^(8)^.

Food-insecure older adults are more likely to consume nutrient-poor, high-energy foods, contributing to obesity and other health challenges^(9,10)^. FIS is also linked to reduced dietary diversity, often favouring high-energy foods over nutrient-dense foods such as vegetables, eggs, legumes, seeds, dairy products, fruits and nuts^(11,12)^. This nutritional imbalance further elevates the risk of obesity^(13)^. Moreover, unlike younger individuals, the elderly people face a complex interplay of aging-related, psychological, social and economic factors that influence their nutritional and health status. For example, conditions such as sarcopenia, frailty and chronic illnesses are more prevalent in this demographic. FIS has been considered as a potent risk factor for sarcopenic obesity^(14)^. However, some studies revealed that weight is not dependently associated with FIS in the elderly^(15,16)^. This controversy results show that various covariates may influence weight status and contribute cumulatively to the likelihood of experiencing FIS.

The National Health and Nutrition Examination Survey (NHANES) from 2017 to 2020 provides an unparalleled opportunity to explore the association between household FIS with diet quality and body composition among older US adults. The current study aims to fill an important gap in the literature by investigating the relationship between FIS status and diet quality as assessed by HEI-2020 and adherence to the MedDiet. Moreover, to the best of our knowledge, no studies have specifically examined the association between FIS and abdominal obesity indices among elderly populations. This study aims to elucidate how FIS impacts body composition, with a focus on A Body Shape Index (ABSI), obesity status, the waist-to-hip ratio (WHR), the waist-to-height ratio (WHtR), Conicity index (as an indicator of abdominal obesity determined using measurements of body mass, height and waist circumference)^(17)^ and abdominal volume index (AVI).

## Methods

### Study design and data source

This research employed a cross-sectional study design to investigate the relationship between FIS and various measures of diet quality and body composition among older adults in the USA. The data for this study were derived from the National Health and Nutrition Examination Survey (NHANES) for the years 2017 to 2020, available for public use at https://wwwn.cdc.gov/nchs/nhanes. NHANES is a comprehensive program conducted by the Centers for Disease Control and Prevention, combining interview data, physical examinations and laboratory tests to assess the health and nutritional status of the US population. This dataset is nationally representative, collected through a stratified, multistage probability sampling design that ensures broad demographic and geographic representation. NHANES was selected because of its robust methodology, detailed nutritional assessments and reliable measures of food security and anthropometric indices.

The procedures, protocols, questionnaires, consent forms and participant information for NHANES were rigorously reviewed and approved by both the Office of Management and Budget and the Ethics Review Board of the National Center for Health Statistics. Ethical oversight ensured adherence to federal standards for protecting human research participants. In addition, written informed consent was obtained from all participants before their enrolment in the study. This process ensured that participants were thoroughly informed about the objectives of the study, procedures and potential risks or benefits, in line with established ethical guidelines^(18)^.

### Study population

**Figure 1.**
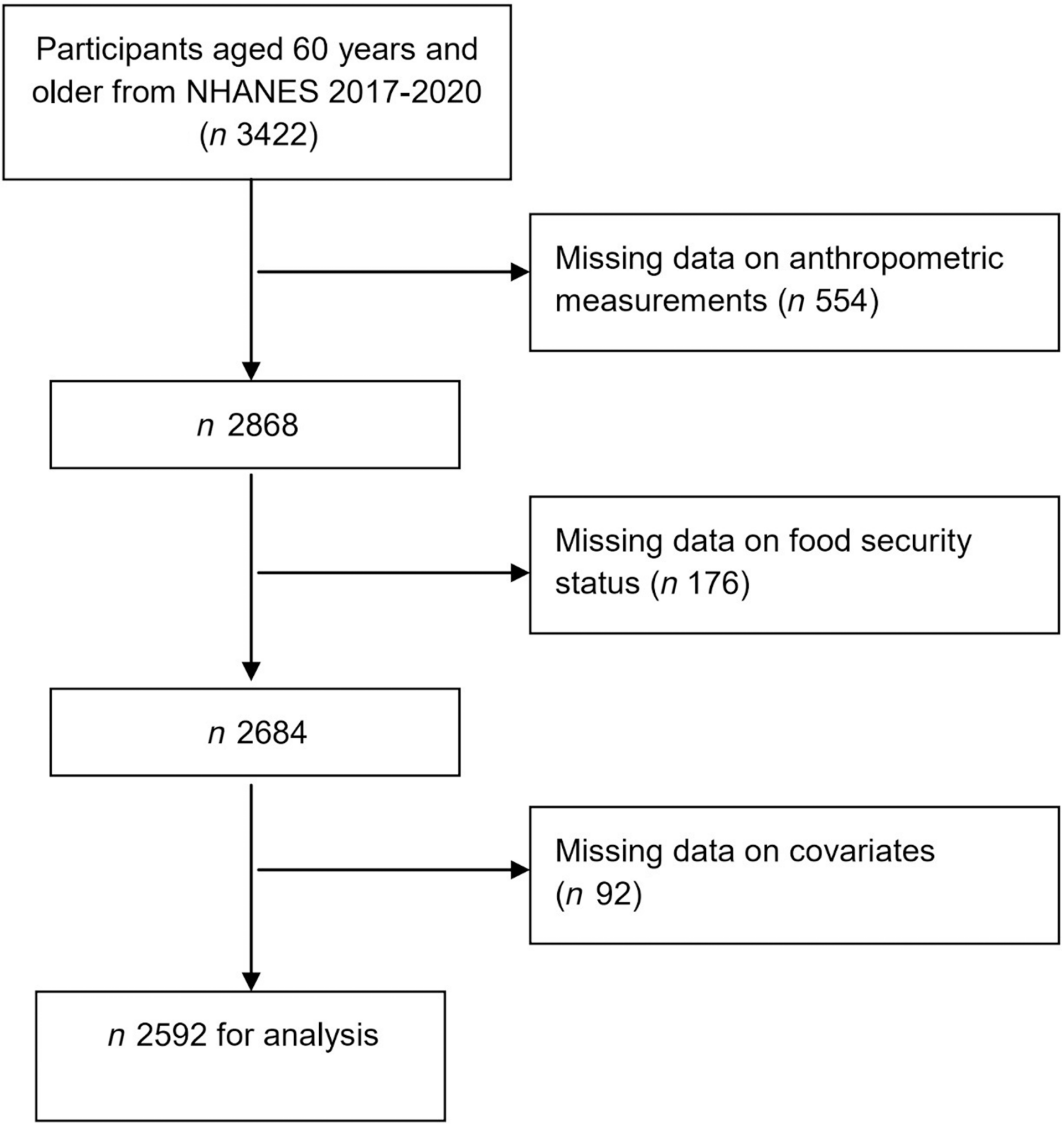
Flowchart of participant selection from NHANES 2017 to 2020.

The study focussed on individuals aged 60 years and older to specifically address the unique vulnerabilities of the elderly population, particularly concerning FIS and its health implications. Inclusion criteria required complete data for household food security status, diet quality indices and anthropometric measurements. Furthermore, patients with missing data including race, education level, income level and physical activity status were excluded. Ultimately, a total of 2592 participants were included in this study. Further specific details are available in Figure 1.

#### Assessment of food security

Food security was assessed using the US Department of Agriculture’s Household Food Security Survey Module, a validated instrument designed to measure the adequacy of household access to food over the past 12 months. This module includes eighteen items addressing financial constraints, food availability and experiences of hunger or reduced dietary intake. As no child was included in the analysis, the focus was on ten items related to the adults^(1)^. Based on responses, households were classified as either food secure (FS) (0–2 affirmative responses categorised as fully or marginal FS), indicating consistent access to adequate food or food insecure (FIS) (3–10 affirmative responses categorised as low and very low FS), signifying limited or uncertain access due to financial or logistical barriers. This dichotomous classification enabled clear differentiation between the two groups and facilitated a detailed analysis of the impact of FIS on health outcomes.

### Diet quality assessment

Dietary intake was evaluated through two 24-hour dietary recalls conducted by trained interviewers. The initial recall occurred in person at the Mobile Examination Center, followed by a second recall via telephone 3–10 d later. These recalls gathered comprehensive information on all foods and beverages consumed from midnight to midnight of the preceding day, including details such as consumption times, eating occasions and locations. Dietary recalls were collected on both weekdays and weekends. The validity of the 24-hour recall method has been previously established^(19,20)^.

For this analysis, we focused on two diet quality indices: HEI-2020 and MedDiet adherence score. The HEI-2020 is a measure of diet quality developed by the US Department of Agriculture Centre for Nutrition Policy and Promotion that measures adherence to the 2020–2025 Dietary Guidelines for Americans^(8)^. The HEI-2020 includes thirteen components: whole fruits, total fruits, greens and beans, total vegetables, dairy, whole grains, total protein foods, plant proteins and seafood, refined grains, fatty acids, saturated fats, added sugars and Na. Each component is scored to reflect adherence, with a maximum total score of 100 points, indicating optimal diet quality^(8)^. The Food Patterns Equivalents Database was utilised to convert reported food and beverage intakes into the necessary food group and nutrient equivalents required for HEI-2020 scoring. Each participant’s HEI-2020 score was calculated by comparing their dietary intake to the standards set for each component. The HEI-2020 scores were calculated using the simple HEI scoring algorithm, which involves deriving ratios of dietary components per 1000 calories and scoring them according to established standards (https://epi.grants.cancer.gov/hei/calculating-hei-scores.html).

Adherence to the MedDiet was quantified by determining intakes of alcohol, red and processed meat, seafood, whole grains, legumes, nuts, fruits, vegetables (excluding potatoes) and the ratio of monounsaturated to saturated fatty acids. One point was assigned to participants who had an intake of these components (except for alcohol and red/processed meat) higher than the median intake for the study population. For alcohol, participants were awarded one point for moderate alcohol consumption defined as 10–25 g/d for men and 5–15 g/d for women. Similarly, for red and processed meats, one point was assigned to those whose intake was below the median for the population. Participants who did not meet these criteria for each component received zero points. The maximum possible score was 9, with higher scores indicating better adherence to the MedDiet^(21,22)^.

### Anthropometric measurement

**Figure 2.**
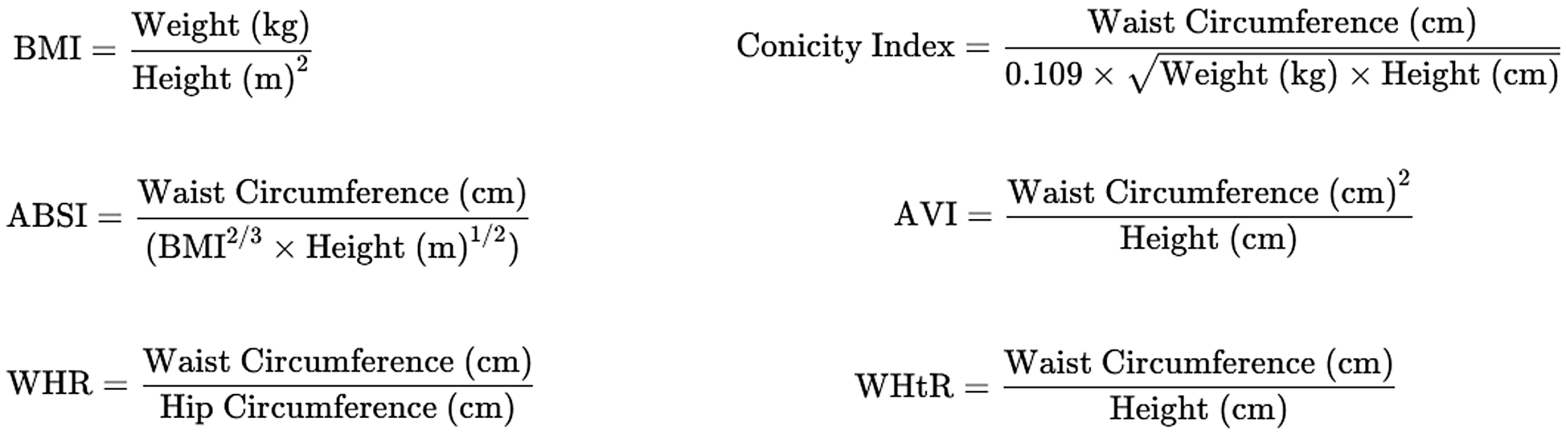
Calculation methods for anthropometric measures.

Anthropometric data were collected following standardised NHANES protocols, ensuring consistency and accuracy. The measures included BMI to provide a general measure of adiposity, ABSI to capture body shape and central obesity, WHR and WHtR to assess fat distribution and abdominal obesity, the Conicity index to quantify abdominal obesity and the AVI to offer a volumetric perspective on central obesity. BMI was categorised as normal (18·5–24·9 kg/m^2^), overweight (25–29·9 kg/m^2^) and obesity (≥ 30 kg/m^2^)^(23)^. Calculation methods are shown in Figure 2^(24)^.

### Covariates

To account for confounding factors, a range of demographic, socio-economic and behavioural variables were included as covariates in the analysis. Demographic factors included age, sex and race/ethnicity, which are known to influence both food security and body composition. Socio-economic factors such as education level, marital status and the family income-to-poverty ratio (FIPR) were incorporated to capture the economic and social dimensions of the lives of participants. FIPR was determined according to the guidelines provided by the Department of Health and Human Services. In the NHANES dataset, FIPR values range from 0 to 5, with a value of 1 signifying 100 % of the federal poverty threshold. For analysis purposes, the FIPR was categorised into three groups: ≤ 130 %, 130 % < FIPR ≤ 349 % and > 350 %. A higher FIPR indicates a higher income level within the family^(25,26)^. The mean energy intake of subjects was measured from two 24-hour dietary recall interviews conducted by trained interviewers. Physical activity was assessed using the NHANES physical activity questionnaire, which follows the structure of the Global Physical Activity Questionnaire^(27)^. The total metabolic equivalent (MET) score was determined as follows: physical activity (MET score) = sum of (days per week of each activity × duration of each activity (min) × corresponding MET value). For vigorous and moderate recreational and occupational activities, 8 and 4 MET were considered, respectively. The final MET scores were then grouped into four levels of activity: inactive (MET min/week < 250), somewhat active (250 ≤ MET min/week < 500), active (500 ≤ MET min/week < 1000) and very active (MET min/week ≥ 1000)^(28)^.

### Statistical analysis

Descriptive statistics summarised the demographic, socio-economic and anthropometric characteristics of the study population, providing a detailed profile of participants based on their food security status. Continuous variables, such as age and BMI, were compared between FS and food insecure groups using independent *t* tests, while categorical variables, like race/ethnicity and marital status, were analysed using chi-square tests. To examine the association between FIS with diet quality and anthropometric indices, multivariable logistic regression models were used, evaluating the likelihood of food insecure individuals falling into the higher quartile of anthropometric measures and the tertile of diet quality indices. In our study, we categorised anthropometric measures into quartiles and diet quality indices into tertiles, following approaches commonly used in previous NHANES-based studies. Both unadjusted and adjusted models were constructed, with the adjusted models controlling for demographic, socio-economic and behavioural covariates. The results were expressed as OR with 95 % CI. Statistical significance was set at *P* < 0·05. All analyses were performed using SPSS software (V25; SPSS Inc.).

## Results

### Characteristics of subjects

A total of 2592 participants aged 60 years and older (48·5 % women) with complete data were eligible for analysis. Of these, 27·4 % experienced FIS. Table 1 details the demographic characteristics and anthropometric indices of participants based on their food security status. FS participants had a higher average age (69·9 ± 6·86) compared with food insecure participants (66·93 ± 5·85) (*P* = 0·001). Conversely, food-insecure individuals had a significantly higher average BMI (30·22 ± 6·99) than FS individuals (29·59 ± 6·04) (*P* = 0·05). Participants experiencing FIS comprised a higher percentage of Hispanic individuals (32·5 %) and a lower percentage of non-Hispanic Whites (25·5 %).

**Table 1.** Characteristics of the participants depending on the situation of food security or insecurity in the household

Variable	Food secure		Food insecure		*P*-value
Gender					
Male	1090	51·5	245	51·6	0·97
Female	1027	48·5	230	48·4	
Age (years)	69·9	6·86	66·93	5·85	0·001
Weight (kg)	81·4	19·06	82·21	20·33	0·41
BMI (kg/m^2^)	29·59	6·04	30·22	6·99	0·05
WC (cm)	103·4	14·4	104·45	15·63	0·15
Energy intake (KJ/day)	466.49	177.1	431.17	208.72	0·005
Race					0·001
Hispanic	327	21·9	148	32·5	
Non-Hispanic white	1005	47·5	116	25·5	
Non-Hispanic Black	523	24·7	167	36·7	
Non-Hispanic Asian	197	9·3	24	5·3	
Obesity status					0·58
Normal	873	22·2	108	22·7	
Overweight	228	36·6	162	34·1	
Obese	323	41·2	205	43·2	
Education level					0·001
< High school	220	16·2	156	29·5	
High school	747	54·8	336	63·6	
College graduate ≤	395	29	36	6·8	
Marital status					0·001
Married	1276	60·4	189	39·9	
Widowed/divorced/separated	735	34·7	237	50	
Never married	102	4·8	48	10·1	
Income (%FIPR)					0·001
< 130 %	373	18·5	273	62·8	
130–349 %	342	16·9	84	19·3	
≥ 350 %	1303	64·6	78	17·9	
Physical activity status					0·056
Inactive	799	37·8	210	44·6	
Somewhat active	154	7·3	27	5·7	
Active	233	11	46	9·8	
Very active	926	43·8	188	39·9	

Abbreviations: FIPR, family income to poverty level ratio; NHANES, National Health and Nutrition Examination Survey; WC, waist circumference. All continuous variables are presented as mean and standard deviation, and categorical variables are presented as numbers and percentages.

### Association between food insecurity and diet quality indices

Table 2 displays OR and 95 % CI for the relationship between FIS and adherence to MedDiet and HEI-2020 scores among older adults. The analysis was conducted using both crude and adjusted models. Individuals with FIS demonstrated significantly lower odds of adherence to a higher MedDiet score in both Model 1 and Model 2. In Model 1, the odds of adherence to moderate MedDiet levels (T2 *v*. T1) were lower (OR: 0·75, 95 % CI: 0·55, 1·05), though not statistically significant. However, participants from the FIS group had significantly lower odds of adherence to the highest MedDiet tertile (T3 *v*. T1: OR: 0·49, 95 % CI: 0·32, 0·73). Similarly, FIS was associated with lower odds of adherence to higher HEI-2020 scores. Individuals in FIS had lower odds of being in T2 (T2 *v*. T1: OR: 0·75, 95 % CI: 0·58, 0·96) and T3 (T3 *v*. T1: OR: 0·64, 95 % CI: 0·45, 0·91) of HEI-2020 adherence. In Model 2, which included additional covariate adjustments, individuals in FIS had lower odds of adherence to moderate (T2 *v*. T1: OR: 0·72, 95 % CI: 0·54, 0·96) and high (T3 *v*. T1: OR: 0·48, 95 % CI: 0·31, 0·67) MedDiet adherence. Likewise, adherence to HEI-2020 remained significantly lower among individuals in FIS in T2 (T2 *v*. T1: OR: 0·69, 95 % CI: 0·50, 0·95) and T3 (T3 *v*. T1: OR: 0·61, 95 % CI: 0·37, 0·84).

**Table 2.** Association of household food insecurity and adherence to the Mediterranean diet and the HEI-2020 score, 2017–2020 NHANES (OR and 95 % CI)

Diet Quality Index (comparison)	Model	OR	95 % CI
MedDiet (T2 *v*. T1)	Model 1	0·75	0·55, 1·05
	Model 2	0·72	0·54, 0·96
MedDiet (T3 *v*. T1)	Model 1	0·49	0·32, 0·73
	Model 2	0·48	0·31, 0·67
HEI-2020 (T2 *v*. T1)	Model 1	0·75	0·58, 0·96
	Model 2	0·69	0·50, 0·95
HEI-2020 (T3 *v*. T1)	Model 1	0·64	0·45, 0·91
	Model 2	0·61	0·37, 0·84

Abbreviations: NHANES, National Health and Nutrition Examination Survey; MedDiet, Mediterranean diet; HEI-2020, Healthy Eating Index. Model 1 = unadjusted model; Model 2 = adjusted for age, sex, race, physical activity status, household income, marital and education status.

### Association between food insecurity and anthropometric indices

Table 3 displays the association between FIS and the risk of anthropometric indices in both unadjusted and adjusted models. Individuals facing FIS were more likely to fall into the higher quartiles of the ABSI, particularly in Q3 (OR: 1·32, 95 % CI: 1·00, 1·74) and Q4 (OR: 1·10, 95 % CI: 0·83, 1·46). Similarly, FIS was associated with higher WHR. In the unadjusted model, FIS was associated with being in the higher WHR quartiles, specifically Q3 (OR: 1·22, 95 % CI: 0·89, 1·67) and Q4 (OR: 1·33, 95 % CI: 0·99, 1·77). In the adjusted model, the association remained significant for individuals in Q4 (OR: 1·44, 95 % CI: 1·01, 2·06). Furthermore, FIS was associated with an increased likelihood of being in the higher quartiles of the Conicity Index.

**Table 3.** Association of household food insecurity with risk of higher anthropometric indices among study participants, 2017–2020 NHANES (OR and 95 % CI)

Variable	Food insecure			
	Unadjusted		Adjusted	
	OR	95 % CI	OR	95 % CI
Obesity status				
Normal	1		1	
Overweight	0·91	0·69, 1·19	0·81	0·58, 1·12
Obese	1·02	0·79, 1·32	0·82	0·6, 1·13
ABSI				
Q1	1		1	
Q2	0·97	0·73, 1·31	1·02	0·72, 1·43
Q3	**1·32**	**1**, **1·74**	**1·44**	**1·06**, **1·95**
Q4	1·1	0·83, 1·46	**1·46**	**1·07**, **2·01**
WHR				
Q1	1		1	
Q2	1·1	0·82, 1·48	1·05	0·77, 1·44
Q3	1·22	0·89, 1·67	1·28	0·9, 1·81
Q4	1·33	0·99, 1·77	**1·44**	**1·01**, **2·06**
WHtR				
Q1	1		1	
Q2	0·89	0·66, 1·21	0·94	0·68, 1·28
Q3	1·14	0·85, 1·52	1·21	0·89, 1·65
Q4	1·28	0·96, 1·7	1·17	0·86, 1·59
Conicity index				
Q1	1		1	
Q2	0·93	0·7, 1·25	1	0·74, 1·34
Q3	1·09	0·82, 1·45	1·26	0·94, 1·69
Q4	1·09	0·082, 1·43	**1·36**	**1·02**, **1·81**
AVI				
Q1	1		1	
Q2	0·95	0·72, 1·27	0·93	0·69, 1·27
Q3	1·03	0·78, 1·37	1·02	0·75, 1·38
Q4	1·08	0·82, 1·43	0·93	0·69, 1·26

Abbreviations: ABSI, A body shape index; WHR, waist-to-hip ratio; WHtR, waist-to-height ratio; AVI, abdominal volume index. Model 1: crude model. Model 2: adjusted for age, sex, race, physical activity status, household income, marital and education status. Bold values are statistically significant.

## Discussion

In this nationally representative sample of US older adults from the 2017–2020 NHANES, we found that older adults experiencing FIS have significantly lower odds of adhering to higher levels of both the MedDiet and the HEI-2020. Previous studies investigating the association between FIS and diet quality have reported mixed findings. For example, a study using NHANES 2007–2016 data observed that FIS was associated with lower diet quality, as measured by HEI-2015, AHEI-2010 and MedDiet scores, among adults aged 65 and older^(6)^. Similarly, Leung *et al.* reported a negative association between FIS and HEI-2005 scores among lower-income US adults^(29)^. However, other studies have found inconsistent results. For instance, an analysis of NHANES 2011–2014 data suggested that in certain racial groups, food security status was not significantly associated with diet quality based on HEI-2015 scores^(30)^. In addition, Gamba *et al.* reported no association between FIS and diet quality assessed by the AHEI modified for pregnancy in NHANES 1999–2008^(31)^. Our study extends these findings by specifically examining adherence to the MedDiet and HEI-2020. The MedDiet is renowned for its health benefits, including reduced risks of cardiovascular and neurodegenerative diseases, as well as cancer^(32)^. The HEI-2020 measures adherence to the Dietary Guidelines for Americans, with higher scores indicating better diet quality. The observed lower adherence among food-insecure older adults suggests that FIS may impede the ability to maintain dietary patterns associated with positive health outcomes. Therefore, addressing FIS in this population is crucial, as it may improve diet quality and, consequently, health outcomes.

The association between FIS and anthropometric indices in our study yielded mixed yet noteworthy findings. While no significant relationship was observed between FIS and general overweight or obesity status based on BMI, FIS was significantly associated with higher values in alternative measures of abdominal obesity, including ABSI, WHR and Conicity index after adjusting for confounding factors. This association suggested that FIS individuals are at greater risk of central fat accumulation^(33)^. To the best of our knowledge, no studies have specifically examined the association between FIS and abdominal obesity indices among elderly populations. However, Rezaei *et al.* explored the relationship between FIS and various body shape indices, including ABSI and the body roundness index, in a US adult population. Their results indicated that FIS was linked to higher odds of elevated ABSI and body roundness index, suggesting an association between FIS and central adiposity. Interestingly, unlike our findings, their study reported a significant association between FIS and BMI, which might be attributed to the inclusion of younger age groups in their analysis^(34)^. Similarly, another study reported that FIS was positively associated with WHR among female participants^(35)^. BMI is a widely used measure to assess general obesity; however, it does not differentiate between lean mass and fat mass, nor does it accurately reflect visceral fat accumulation. While waist circumference is often regarded as the gold standard for evaluating abdominal obesity due to its simplicity and correlation with central fat, it too has limitations in capturing the specific contribution of visceral fat epidemiologically^(33)^. Therefore, alternative indices such as ABSI, WHR and the Conicity Index have been developed to provide a more accurate assessment of abdominal obesity and its associated health risks. Interestingly, although WHtR and AVI are also recognised as central obesity indices, FIS was significantly associated only with ABSI, WHR and the Conicity Index. These indices have some differences. ABSI and the Conicity Index are shape-sensitive indices and are the only abdominal obesity indices not affected by the obesity paradox^(33)^; while WHtR and AVI are size-based indices and can be affected by the obesity paradox^(36,37)^. This paradox suggests that in some individuals with specific conditions, like heart failure, higher BMI or other size-related metrics might be associated with better outcomes^(38)^. Nagayama *et al.* showed that ABSI and the Conicity index had the strongest correlations with age and arterial stiffness than WHtR and waist circumference^(33)^. These findings suggest that FIS may be more closely linked to unfavourable changes in body shape and fat distribution, rather than gross abdominal size, highlighting the importance of using shape-sensitive indices like ABSI and the Conicity Index when assessing metabolic risk in vulnerable populations.

Diet quality could serve as a mediating factor in the association between FIS and central obesity. Research has extensively examined the relationship between adherence to the MedDiet and central obesity. A systematic review of eighteen intervention trials found that 72 % of the studies reported a significant reduction in central obesity measures, among participants following a Mediterranean-type diet. However, it’s noteworthy that seven of these studies included energy restriction, and only three demonstrated a statistically significant favourable effect of the MedDiet compared with control groups^(39)^. The specific relationship between HEI-2020 adherence and central adiposity remains underexplored, indicating a need for further investigation in this area. However, a negative association between other versions of HEI and central obesity was found in some studies^(40)^. A study utilising data from the 2015 to 2018 NHANES found that central obesity significantly mediated the association between HEI-2015 scores and low-grade inflammation-related serum inflammatory markers^(41)^.

Several mechanisms may explain our findings on anthropometric measures. Our observed finding regarding the association between FIS and abdominal obesity, despite a lack of significant differences in BMI, is referred to as the ‘hunger-obesity paradox’. This phenomenon suggests that poor dietary quality among food-insecure individuals may contribute to fat deposition patterns that disproportionately affect central regions^(42)^. While FIS is often associated with reliance on inexpensive, energy-dense and nutrient-poor foods that promote visceral fat accumulation^(43)^, our analysis revealed that the FS group had a significantly higher energy intake than the FIS group. This discrepancy could be attributed to FS individuals having access to a more diverse diet that includes both nutrient-rich and energy-dense options^(44)^, leading to greater overall energy consumption. This lower dietary diversity, coupled with a tendency toward high energy intake from inexpensive, energy-dense foods, may contribute to the increased risk of abdominal obesity observed in the FIS group^(43)^. It should be noted that not all energy-dense diets have the same metabolic effects. Second, the psychological stress associated with FIS may dysregulate cortisol levels^(45)^, promoting central adiposity^(46)^. Finally, limited access to healthcare and preventive services may exacerbate health disparities in food-insecure populations, compounding their vulnerability to adverse anthropometric outcomes^(47)^.

This study benefits from a robust dataset derived from a nationally representative sample, allowing for generalisability to the US older adult population. We utilised the most recent versions of diet quality assessment tools to ensure alignment with current dietary guidelines. Moreover, the use of advanced anthropometric indices such as ABSI and the Conicity Index provides a more comprehensive understanding of adiposity patterns than BMI alone. Additionally, the adjustment for potential confounders strengthens the validity of the findings. Moreover, this is the first study examining the association between FIS with HEI-2020 and abdominal obesity indices among elderly populations. However, certain limitations warrant consideration. First, the cross-sectional nature of the study precludes causal inferences. Longitudinal studies are needed to better understand the temporal relationship between FIS and changes in diet quality and anthropometric indices. Second, the reliance on self-reported dietary and income data may introduce recall or reporting biases. Third, FIS can fluctuate, and a one-time measure may not capture chronic *v*. transient FIS.

### Conclusion

Older adults experiencing FIS have significantly lower odds of adhering to higher levels of both the MedDiet and the HEI-2020. This study underscores the complex relationship between FIS and anthropometric indices among older adults. Although no significant link was found between FIS and general obesity (BMI), the study observed a significant association with central adiposity measures, including ABSI, WHR and the Conicity Index, suggesting that FIS may predispose individuals to central fat accumulation. Future research should explore the longitudinal effects of FIS on diet quality and adiposity patterns and investigate targeted interventions to reduce health disparities in this population.
